# Evaluation of the Usefulness of a Serological Test for Diagnosis of Celiac Disease Simultaneously Detecting Specific Antibodies and Total IgA

**DOI:** 10.3390/nu15010202

**Published:** 2022-12-31

**Authors:** Emilia Majsiak, Bożena Cukrowska, Magdalena Choina, Kornel Bielawski, Joanna Cielecka-Kuszyk, Ewa Konopka, Mariusz Wysokiński, Joanna Beata Bierła

**Affiliations:** 1 Department of Health Promotion, Chair of Nursing Development, Faculty Health of Sciences, Medical University of Lublin, Staszica 4/6, 20-081 Lublin, Poland; 2 Department of Pathomorphology, the Children’s Memorial Health Institute, Aleja Dzieci Polskich 20, 04-730 Warsaw, Poland; 3 Polish-Ukrainian Foundation of Medicine Development, Nałęczowska 14, 20-701 Lublin, Poland; 4 Department of Basic Nursing, Chair of Development in Nursing, Faculty of Health Sciences, Medical University, Staszica 4/6, 20-081 Lublin, Poland

**Keywords:** celiac disease, serological screening, total IgA, IgA deficiency

## Abstract

The diagnosis of celiac disease (CD) at the first diagnostic step requires the detection of specific class A antibodies to tissue transglutaminase type-2 (TG2 IgA) and the measurement of total immunoglobulin A (tIgA) to exclude IgA deficiency. The aim of the study was to evaluate the new quantitative immunoassay panel allowing for the detection of celiac-specific antibodies with the simultaneous determination of tIgA from the same sample of blood at one time. This retrospective study included 104 pediatric patients divided into groups with recognized CD and IgA deficiency (n = 20; 19%), immunocompetent children with CD (n = 28; 27%), children with IgA deficiency and without CD (n = 28; 27%), and the control group of immunocompetent children without CD (n = 28; 27%). Intestinal biopsy with histopathological evaluation (except five patients with CD who were diagnosed without biopsy) and measurement of reference celiac specific antibodies were performed in all children. Multiparametric quantitative immunoassay Polycheck^®^ Celiac IgA plus total IgA test was used to evaluate its usefulness in CD screening and IgA deficiency diagnosis. The statistical analysis showed the high sensitivity and specificity of both TG2 IgA and tIgA on the multiparametric panel (sensitivity 96% and 100%; specificity 100% and 79%, respectively). The accuracy and area under the ROC curve for tIgA were 0.904 and 0.955, while for TG2 IgA they were 0.982 and 1.000, respectively. Although the sensitivity of IgA antibodies against deaminated gliadin peptides was low (20%), the specificity reached 100%. The study showed that Polycheck^®^ Celiac IgA plus total IgA test is a specific and sensitive tool for simultaneous serological CD screening and recognition of IgA deficiency.

## 1. Introduction

Celiac disease (CD) is a chronic, autoimmune disease of the small intestine, which occurs in genetically predisposed individuals due to exposure to gluten [1]. Both genetical and environmental factors are engaged in the development of the disease. The consumption of gluten by CD-prone individuals activates innate and adaptive immune responses leading to the destruction of small intestine mucosa [2,3]. Although symptoms of malabsorption, such as abdominal pain, diarrhea and malnutrition, are most frequently associated with CD, the clinical picture of the disease is changing [4,5,6]. Recently, our study showed that anemia and chronic fatigue were among the most common symptoms reported by Polish CD patients [5]. Thus, the various clinical manifestations of CD may pose a challenge to clinicians, which results in persistent symptoms, reduced patient quality of life and delayed diagnosis [5,7,8].

According to obligatory recommendations of the European Society for Paediatric Gastroenterology, Hepatology and Nutrition (ESPGHAN), the diagnostic procedure for CD should begin with the determination of autoantibodies against intestinal tissue transglutaminase type 2 (TG2) in the immunoglobulin A (IgA) class [9]. Due to the co-occurrence of IgA deficiency in CD (2–8%), determination of total IgA (tIgA) is necessary. When IgA deficiency is recognized, serological test detecting immunoglobulin G (IgG) against TG2 or against deamidated gliadin peptides (DGP) or endomysium (EMA) should be performed followed by histological examination of intestinal biopsies of the small intestine [9,10]. In 2012, ESPGHAN introduced a new possibility of establishing CD diagnosis without intestinal biopsy in children and adolescents with CD symptoms [10], and in 2020, even in those at-risk groups without such symptoms, who have high (>10 times upper limit of normal, ULN) TG2 IgA concentration in sera. However, in such cases, to reduce the risk of laboratory errors, EMA should be determined in another blood sample. In contrast to the ESPGHAN guidelines from 2012, the new ones from 2020 do not recommend performing HLA-typing for the no-biopsy approach in children and adolescents [9].

As CD symptoms may occur at any age, it is worth highlighting that serology is the first step in the CD diagnostic process also in adults. The American Gastroenterological Association recommends beginning the diagnostic process with the detection of TG2 IgA and tIgA. IgA deficiency in adults also requires the detection of TG2 IgG or DGP IgG or EMA IgG. In symptomatic adult patients with high (>10 times ULN) TG2 IgA titers and positive EMA in a second blood sample, esophagogastroduodenoscopy and intestinal biopsy cannot be omitted [11]. However, a discussion has already begun regarding, as in children, the possibility of omitting the biopsy when high TG2 IgA concentrations are present in the sera of adult patients [12].

In patients with CD, we can determine TG2, EMA or DGP IgA and IgG antibodies. The detection of EMA is no longer recommended in the first diagnostic step of CD, because they may be absent in the early stage of the disease. Despite their high specificity, EMA antibodies have lower sensitivity than TG2 antibodies [13]. It is also worth noting that the antigen for both TG2 and EMA antibodies is the same enzyme (tissue transglutaminase 2), but the method of detecting these antibodies differs. EMA antibodies are determined by indirect immunofluorescence, while TG2 antibodies are detected by the following methods: chemiluminescence, immunoblot, enzyme immunoassay (ELISA) and radioimmunoassay (RIA) [10]. Currently, it is recommended to determine EMA antibodies in the IgA class as the second test performed from a separate blood draw in children who meet the criteria for CD diagnosis without biopsy [9].

The determination of antibodies to native gliadin (anti-AGA) is no longer recommended in the diagnostic process of CD due to their low sensitivity and specificity, and they are currently not used [10].

The most sensitive (sensitivity of tests about 99%) and specific (specificity of tests about 98%) markers of CD are TG2 antibodies in the IgA class. In addition to diagnosis, a significant aspect of serological tests is their use to monitor therapy, i.e., the effectiveness of the GFD [14]. The concentration of TG2 IgA or DGP IgG antibodies decreases with strict adherence to the GFD [15]. TG2 autoantibodies in some patients persist for quite a long time (up to several years), while DGP antibodies against the factor-inducing disease processes are a good marker of adherence to a gluten-free diet. A negative result confirms compliance with dietary recommendations [16].

Many authors emphasize the increase in analytical accuracy when determining several markers during one test [17,18]. Previous methods of detecting CD-specific antibodies were based on various enzyme-linked immunoassays, enabling individual measurements of one type of antibody from a given blood sample (singleplex). More recently, multiparameter tests have been introduced to quantify several CD markers in one test (multiplex). One of the tests available on the Polish market is Polycheck^®^ Celiac IgA plus Total IgA, with human recombinant TG2 antigen and DGP peptides, which allows for both the detection of class A antibodies and total IgA. Another test available in Poland is Polycheck^®^ Celiac IgA plus Total IgA and Polycheck^®^ Celiac IgG, which contains human recombinant TG2 antigen, DGP peptides and intrinsic factor IF (for the differential diagnosis of anemia—the main symptom of CD), for the detection of antibodies in the IgG class. Retrospective studies have shown that the performance of both tests allows you to achieve a sensitivity of 98%, a specificity of 100% and a diagnostic value of 99%, assuming that the patient had at least two positive markers out of four (TG2 IgA, DGP IgA, TG2 IgG and DGP IgG). The high usefulness of the Polycheck Celiac IgG panel in the group of patients with selective IgA deficiency was also demonstrated [19].

The aim of the current study was to evaluate the new screening quantitative immunoassay panel for the detection of celiac-specific antibodies with the simultaneous determination of tIgA from the same sample of blood at one time.

## 2. Materials and Methods

### 2.1. Patients and Study Design

This study was retrospective and enrolled 104 pediatric patients treated in the Children’s Memorial Health Institute, Warsaw, in Poland. The children were divided into 4 groups. The first group included children with CD and IgA deficiency (n = 20; 19%). In the second group were immunocompetent children with CD (n = 28; 27%). The third group consisted of children without CD, but with IgA deficiency (n = 28; 27%). The fourth group was the control group, in which were immunocompetent children without CD (n = 28; 27%). The patients’ characteristics are presented in Table 1. All included children except 5 patients with recognized CD underwent intestinal biopsy during endoscopy with histological examination of duodenal specimens. Biopsy results were assessed with the Marsh–Oberhüber scale [20] (Table 2). The changes classified as Marsh 0 corresponded to the normal morphological picture of the mucosa; Marsh 1—to an increase in the number of intraepithelial lymphocytes above 25/100 enterocytes; Marsh 2—to an increase in the number of lymphocytes (>25/100 enterocytes), shortening and flattening of intestinal villi, and crypt hyperplasia; Marsh 3—to an increase in intraepithelial lymphocytosis (>25) and atrophy of intestinal villi. CD was recognized according to the ESPGHAN recommendations (2012, 2020) in patients with histopathological changes described as at least Marsh 2 grade in the Marsh–Oberhüber classification and with the presence of specific TG2 IgA or TG2 IgG/DGP IgG (in case of IgA deficiency) antibodies in sera. Antibodies were detected with the use of a Phadia 100 analyzer and reference EliA commercial kits (Thermo Scientific Phadia GmbH, Freiburg, Germany). In 5 patients with high TG2 IgA (>10 times ULN) concentration, CD was diagnosed without intestinal biopsy. In all of the patients, genetic predisposition to CD (the presence of HLA-DQ2 and/or HLA-DQ8) and the presence of EMA (Euroimmun, Lübeck, Germany) were confirmed. The study was conducted in accordance with the Declaration of Helsinki and approved by the local Ethics Committee of the Children’s Memorial Health Institute (no 62/KBE/2016 and date 14 December 2016). The written informed consent was obtained from all patients (>16 years old) or their parents/guardians with respect to the use of their blood for scientific purposes.

### 2.2. Antibody Determination by Multiparametric Immunoassay

**Figure 1:**
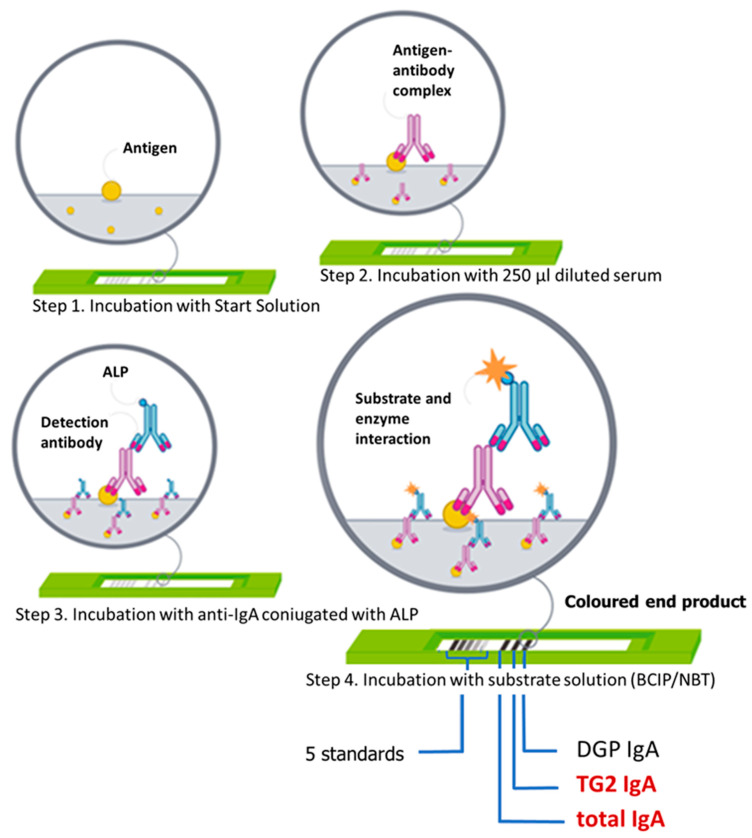
Schematic steps of the multiparametric immunoassay quantitative test Polycheck^®^ Celiac IgA plus total IgA (Biocheck, GmbH, Münster, Germany). BCIP/NBT = 5′bromo-4′chloro-3′indolylphosphate/4′ nitrobluetetrazolium; ALP = alkaline phosphatase.

Specific celiac antibodies and tIgA level were measured with the use of a quantitative, multiparametric immunoassay (Polycheck^®^ Celiac IgA plus total IgA, Biocheck, GmbH, Münster, Germany). The test enables the detection of specific IgA antibodies against human recombinant tissue TG2, DGP and tIgA from the same blood sample at one time. Every test for each patient contains lines of relevant antigens, which are coated together with 5 IgA concentrations (lines) of calibrators on a nitrocellulose membrane located in the cassette. Multiparametric test was done in sera collected from all children. Serum samples were stored at −40 °C until antibodies determination was performed. The test was performed according to the manufacturer’s guidelines. Briefly, after incubation with a start solution (buffered protein solution), two hundred and fifty microliters of patient’s sera was diluted at 1:100 (15 µL + 1485 µL diluent) and incubated for 45 min at room temperature. In the next step, anti-human-IgA monoclonal alkaline phosphatase conjugated detection antibodies were added and incubated for 30 min. After washing, to visualize the reaction, the substrate (5′bromo-4′chloro-3′indolylphosphate/4′ nitro-bluetetrazolium; BCIP/NBT) was added for 20 min. Next, the cassettes were washed and dried in the dark, at room temperature, for approximately 30 min. Then, the color intensity of the specific lines, corresponding to antibody and tIgA concentrations in the patient’s serum, was read with the use of Biocheck Imaging Software, and the results were calculated according to the calibrator curve in each cassette. Thanks to the calibrator curve, the concentration of each antigen-specific IgA as well as tIgA was quantified and reported in International Units (kU/L). According to the manufacturer’s protocol, the value >0.8 kU/L was considered positive for TG2 IgA and DGP IgA. A tIgA value of less than 0.5 kU/L meant IgA deficiency (with gray zone 0.50–0.80 kU/L). The whole procedure lasted for 1 h and 45 min (Figure 1).

### 2.3. Statistical Analysis

The diagnostic performance of Polycheck^®^ Celiac IgA plus total IgA serological test was determined by calculating the sensitivity, specificity, positive and negative predictive values (PPVs and NPVs), accuracy (ACC), areas under receiving operator characteristic curves (AU ROC), likelihood ratios (LR), Youden index and error rate. Receiver operating characteristic (ROC) curves were used to assess the diagnostic predictive capacity of the selected analyzed biomarkers.

The area under the curve (AUC) was calculated in order to estimate the diagnostic accuracy. Data were analyzed using Statistica 12.5 software (StatSoft, Krakow, Poland).

## 3. Results

**Figure 2:**
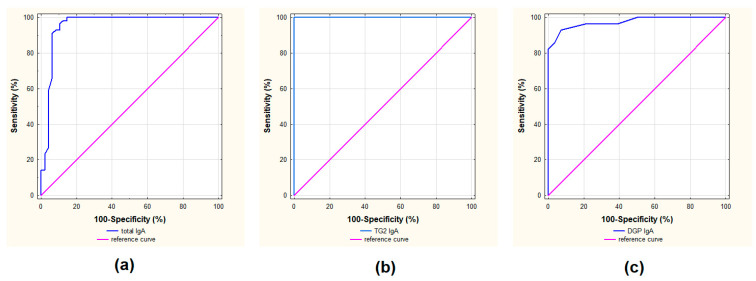
ROC curve and AUC for tIgA (**a**), TG2 IgA (**b**) and DGP IgA (**c**) on the panel Polycheck^®^ Celiac IgA plus total IgA.

In this study, the utility of simultaneous detection of celiac serological antibodies in class IgA and tIgA from the same blood sample was assessed. The sensitivity, specificity, PPV, NPV, AU ROC, likelihood ratios for positive (LR+) and negative (LR−) results, Youden index and error rate for TG2 IgA, DGP IgA and tIgA were determined. The statistical performance of each marker is presented in Table 3 and Figure 2.

The statistical analysis showed the high sensitivity and specificity of both TG2 IgA and tIgA on the panel Polycheck^®^ Celiac IgA plus total IgA. When the cut-off proposed by the manufacturer for tIgA (0.5 kU/L) was chosen, the sensitivity and specificity were 100% and 79%, respectively. The ACC for tIgA was 0.904, and the area under the ROC curve was 0.955 (Figure 2). Meanwhile, the PPV was 0.848, the NPV was 1.000, and likelihood ratios for positive (LR+) and negative (LR−) results were 4.800 and 0.000, respectively. The Youden index was 0.792 and the error rate 0.096.

The sensitivity and the specificity of TG2 IgA regarding the measurement when the CD diagnosis was established were 96% and 100%, respectively. The NPV was 0.966 and the PPV was 1.000. Diagnostic accuracy determined by the AU ROC curve value for TG2 IgA was 0.982. For TG2 IgA, the likelihood negative ratios (LR−) results, Youden index and error rate were 0.036, 0.964 and 0.018, respectively. In contrast to TG2 IgA, the sensitivity of DGP IgA was low reaching a value below 20%, whereas the specificity was 100%. This section may be divided by subheadings. It should provide a concise and precise description of the experimental results, their interpretation, as well as the experimental conclusions that can be drawn.

## 4. Discussion

The principal serological marker in the diagnostic process of CD is TG2 antibodies in class A (when IgA deficiency is recognized, the measurement of antibodies in G class should be performed). If TG2 antibodies are present, the authenticity of CD origin is above 98%. Once we detect TG2 IgA on a panel test, other celiac antibodies do not need to be determined (even if TG2 IgA are present, there is a possibility that we will not detect antibodies in class G) [10]. If IgA deficiency has been recognized, antibodies in class G should be measured—TG2 and DGP. When a patient does not have TG2 IgA, but has appropriate IgA titers and positive DGP, which are not specific markers of CD, the intestinal biopsy should be considered, regarding clinical symptoms. When no lesions are detected in the biopsy, the symptoms should be observed, and after 6–12 months, antibodies need to be measured again (preferably with the same test to have a reference point).

When only DGP are present, without other celiac markers and changes typical of CD in the biopsy, other causes of the presence of DGP should also be taken into account, e.g., non-celiac gluten sensitivity [16].

Due to the higher prevalence of IgA deficiency in people with CD (2–8%) than in the general population (0.25%) [21], the ESPGHAN 2012 and 2020 guidelines recommend simultaneous detection of TG2 IgA with tIgA [9,10]. The detection of tIgA enables the identification of patients with IgA deficiency, which determines the following steps in the CD diagnosing process. When IgA deficiency is recognized, specific IgG antibodies should be detected, and then small intestine biopsies should be taken for histological examination. Thus, the current study presents the usefulness of a serological test simultaneously detecting specific celiac antibodies and tIgA (the panel Polycheck^®^ Celiac IgA plus total IgA) from the same sample of blood at one time.

Recently, a limited number of strategies using different combinations of tests detecting more than a single celiac-specific antibody simultaneously have been developed to achieve better clinical efficacy of CD [22]. The previous version of the Polycheck^®^ Celiac IgA assay included detection only of TG2 IgA and DGP IgA without determination of tIgA. In a retrospective study, the sensitivity of these antibodies was 97.7% and 34.1%, respectively, whereas the specificity 100% and 98%, respectively [19]. In order to achieve better clinical performance of this multiparametric immunoassay, the measurement of tIgA, together with TG2 IgA and DGP IgA, was introduced. Multiparameter tests may lead to lower sensitivity in exchange for higher specificity [18]. Nevertheless, in this study, the multiparameter strategy still achieved high sensitivity (96%). The new panel (Polycheck^®^ Celiac IgA plus total IgA) has enabled the diagnosis of both CD and IgA deficiency in one diagnostic step. The simultaneous detection of several antibodies in the blood sample enabled us to view their performance. Each panel measures celiac-specific IgA or IgG antibodies from a single serum sample, which is a novel approach that does not use a combination of separate ELISA performances.

The whole medical market, including the diagnostics sector, is searching for the most effective solutions, both in clinical and financial aspects [23]. The development and use of multiparameter immunoassays that can simultaneously quantify many different autoantibodies seem to be particularly beneficial regarding various immunological diseases. In his work, Damoiseaux J. (2016) shows that multi-parameter methods of detecting antibodies in systemic autoimmune diseases, but also those limited to organs, seem to be more and more suitable for diagnosing these diseases. He mentions the following examples of the disorders that require the detection of many autoantibodies: myasthenia gravis, pernicious anemia, primary biliary cirrhosis, paraneoplastic neurological syndrome, Guillain–Barré syndrome or Miller-Fisher syndrome. Patients who suffer from autoimmune diseases of the gastrointestinal tract may also benefit from the multiparametric detection of autoantibodies. These diseases include autoimmune gastritis, pernicious anemia, autoimmune hepatitis, primary biliary cirrhosis and CD [24]. Presented in the current study, the simultaneous detection of serological CD markers with tIgA can reduce not only the costs of serological CD diagnosis compared to single celiac-specific antibody detection and tIgA but may also shorten CD diagnostic process. The determination of TG2 IgA and tIgA concentration in patient’s serum with the use of Polycheck^®^ Celiac IgA plus total IgA takes less than two hours, so this method is not time-consuming. It is also worth highlighting that only 250 µL of serum is necessary to perform the test, which is important especially in pediatric patients. Thus, in case of a high concentration of TG2 IgA (>10 times ULN), this small amount of serum is enough to establish CD diagnosis in children (according to the ESPGHAN guidelines in these pediatric patients, an intestinal biopsy can be omitted) [9].

According to the ESPGHAN guidelines, the preferred method for clinical evaluation in CD diagnostics are commercial tests, which use a calibration curve with antibody dilutions providing numerical values proportional to antibody concentration in relative (arbitrary) units [10]. As results obtained with the use of Polycheck^®^ Celiac IgA plus total IgA are read from the 5-point real-time individual calibration curve with reaction background for every tested sample, and expressed in kU/L, the test fully meets these criteria and allows the quantitative results of tested celiac-specific antibodies as well as tIgA to be received.

The panel of Polycheck^®^ Celiac IgA plus total IgA contains not only TG2 for testing TG2 IgA antibodies, but also DGP for testing antibodies against the CD inducer, i.e., gluten peptides. The measurement of DGP IgA or IgG is important when monitoring the GFD. The antibodies’ titers decrease or their absence in control tests confirms the lack of gluten in the diet, even when TG2 are present [1]. We have shown that despite high specificity, DGP IgA have relatively low sensitivity compared to TG2 IgA antibodies (100% and 18%, respectively) in our study group, where the mean age was 10.4 years (ranging from 1.8 to 17.8). Although the latest ESPGHAN guidelines from 2020 do not recommend the use of DGP IgA for CD diagnostics even in children under 2 years of age [9], previous ESPGHAN recommendations from 2012 indicated such possibility [10]. The importance of assessing DGP IgA in the youngest children is still under investigation. Recent studies have shown that DGP IgA provide both high PPV and specificity for CD in children younger than 3 years old [25]. The authors recommend the use of DGP IgA in conjunction with TG2 IgA as CD diagnostic tool in this age.

An isolated positivity for DGP IgA and/or DGP IgG in patients at low risk for CD is predictive of CD in only 15% of cases [26]. The diagnostic accuracy of TG2 IgG was better than IgA DGP, which is concordant with previously made meta-analysis [19]. In our study group there were no isolated positive results for DGP IgA (no positive results for cut-off >0.8 kU/L) when TG2 IgA were absent. In the analyzed group these antibodies were always accompanied by the positive results for TG2 IgA. The comparison of the positive DGP IgA to the results of the reference method revealed that in the presence of DGP IgA on the Polycheck^®^ Celiac IgA plus total IgA panel, the EliA results (Thermo Scientific Phadia GmbH, Freiburg, Germany) were >100 kU/L in all cases. The highest sensitivity and specificity (98% and 100%, respectively) of celiac-specific antibodies were reached in our previous study [19] when we obtained at least two positive results testing four markers (TG2 IgA, TG2 IgG, DGP IgA, DGP IgG). In such cases, the PPV was 100%, the NPV 98% and AU ROC 0.990.

It should also be emphasized that DGP IgA has been successfully used to monitor a gluten-free diet, and DGP antibodies show higher sensitivity than TG2 IgA in monitoring compliance with dietary treatment [27].

The panel of Polycheck^®^ Celiac IgA plus total IgA is the first immunoenzyme screening assay providing the unique opportunity to combine detection of TG2 IgA, DGP IgA, and tIgA. It is worth noting that the test we analyzed provided quantitative results, which is beneficial for diagnosis, follow-up and therapy of CD and many other autoimmune diseases. It is even more important that not all multiparametric immunoassays provide us with quantitative results [24]. An example of immunofluorassay used to detect simultaneously celiac-specific antibodies and tIgA, such as Polycheck^®^ Celiac IgA plus Total IgA, is CytoBead CeliAK (GA Generic Assays GmbH, Dahlewitz, Germany), which is performed with fluorescent microparticles for antigen and antibody immobilization, and monkey-esophagus tissue. Although the results of CytoBead CeliAK can be interpreted both visually by classical fluorescent microscopy and digitally, they cannot be read automated when insufficient number of beads are filled. Grossmann et al. performed a study evaluating clinical usefulness of CytoBead CeliAK on a large study group (380 patients and controls in total, including 155 patients with CD). Nevertheless, the study group included only five patients with IgA deficiency. The authors of the study revealed no significant discrepancies between sensitivity and specificity of the analyzed method and ELISA, when at least one celiac-specific antibody was positive on CytoBead CeliAK. The specificity of TG2 IgA on CytoBead CeliAK was 97.4% when read automatically and increased to 99.4% when read visually, but it was worse compared to specificity of TG2 IgA received with Polycheck^®^ Celiac IgA plus Total IgA in our analysis (100%). Regarding tIgA, the specificity and the sensitivity in our analysis were 79% and 100%, respectively, whereas Grossmann et al. demonstrated that all IgA deficient samples (n = 5) were scored negative by both visual and automatic evaluation. On the other hand, in 374 out of 375 samples from immunocompetent patients, IgA levels were above 0.2 g/L (sufficient) when read automatically and visually [22].

CytoBead CeliAK test and Polycheck^®^ Celiac IgA plus Total IgA present a similar approach to detect various types of IgA antibodies and tIgA simultaneously. Nonetheless, the mentioned assays implement a different technique—an immunofluorassay [22]. Despite this modern approach and good results, CytoBead CeliAK remains an indirect immunofluorassay, and the limitations of this method cannot be omitted. As we know, the preferred method to detect TG2 IgA is ELISA instead of immunofluorassay, as the reading of classical immunofluorassay may be subjective and it may depend on the substrate that was used [22]. However, it is worth highlighting that there are no studies comparing Polycheck^®^ Celiac IgA plus Total IgA and CytoBead CeliAK to the reference method.

This study also has limitations that should be mentioned. The study is retrospective, and prospective studies using this test are necessary to draw conclusions about their usefulness in everyday clinical practice. Another limitation of this study that should be mentioned is the limitations of immunochemical methods.

## 5. Conclusions

The current study shows that the new Polycheck^®^ Celiac IgA plus total IgA test is a useful tool for serological CD screening and recognition of IgA deficiency. The test allows the detection of both specific TG2 IgA and tIgA, i.e., parameters recommended by ESPGHAN as a first diagnostic step in CD recognition, from the same blood sample at one time. Nevertheless, it is essential to continue studies on the diagnostic accuracy of analyzed immunoassay in everyday clinical practice.

## Figures and Tables

**Table 1:** Patients’ characteristics.

	CD Patients		No CD Patients	
	48 (46%)		56 (54%)	
	CD with IgA Deficiency	CD without IgA Deficiency	Non-CD with IgA Deficiency	Non-CD and no IgA Deficiency
Number of patients	20 (19%)	28 (27%)	28 (27%)	28 (27%)
Females	7 (35%)	16 (57%)	16 (57%)	15 (54%)
Males	13 (65%)	12 (43%)	12 (43%)	13 (46%)
Mean age in years (median)	10.3 (10.00)	9.2 (8.7)	10.2 (8.7)	11.9 (13.4)

**Table 2:** The histological changes of intestinal biopsies *.

	CD Patients		No CD Patients	
	CD with IgA Deficiency (n = 20)	CD without IgA Deficiency (n = 28)	Non-CD with IgA Deficiency (n = 28)	Non-CD and no IgA Deficiency (n = 28)
Marsh 0	0	0	28 (100%)	28 (100%)
Marsh II	2 (10%)	0	0	0
Marsh III	18 (90%)	23 (82%)	0	0
CD diagnosis without biopsy	0	5 (18%)	0	0

* Histological changes in small intestinal biopsies were assessed according to the Marsh–Oberhüber classification [20] described in Material and Methods. There were no biopsies classified as Marsh 1.

**Table 3:** The diagnostic assessment of simultaneous detection of celiac specific antibodies and total IgA.

	Cut-Off in kU/L	Sensitivity	Specificity	PPV	NPV	ACC	LR+	LR−	Youden Index	Error Rate
TG2 IgA	0.8	96%	100%	1.000	0.966	0.982	-	0.036	0.964	0.018
DGP IgA	0.8	18%	100%	1.000	0.549	0.589	-	0.821	0.179	0.411
tIgA	0.5	100%	79%	0.848	1.000	0.904	4.800	0.000	0.792	0.096

TG2 IgA, DGP IgA and tIgA were detected simultaneously with the use of Polycheck^®^ Celiac IgA plus total IgA (Biocheck GmBH, Münster, Germany).

## Data Availability

The data presented in this study are available on request from the corresponding author.
